# Application of hybrid cranioendoscopic approaches in anterior skull base surgery

**DOI:** 10.3171/2020.4.FocusVid.19913

**Published:** 2020-04-01

**Authors:** Evan Joyce, Michael Karsy, Serge Makarenko, Gretchen M. Oakley, William T. Couldwell

**Affiliations:** 1 Department of Neurosurgery, Clinical Neurosciences Center, University of Utah; and; 2 Division of Otolaryngology, University of Utah, Salt Lake City, Utah

**Keywords:** anterior skull base, meningioma, microsurgical, endoscopic, cranioendoscopic, video

## Abstract

Anterior skull base approaches have included endoscopic or open microsurgical approaches for intracranial pathologies. However, discussion of a combined hybrid, cranioendoscopic approach, leveraging the benefits of both techniques, has been limited. Here we describe a case of a combined endoscopic, endonasal, and open microsurgical frontotemporal approach for resection of a complex anterior skull base lesion. A 62-year-old man with a large meningioma extending intradurally through the cribiform plate and sphenoethmoidal sinuses underwent a cranioendoscopic resection. Surgical techniques, including repair of the anterior skull base defect as well as complication avoidance and the coordination of multiple surgeons, are discussed.

The video can be found here: https://youtu.be/Ti9tUUdWgJc.

## Transcript

### 0:25 Case

This case demonstrates a 62-year-old man who presented with epistaxis and a nasal mass which was biopsied by an outside ear, nose, and throat specialist and found to be meningioma.

### 0:37 Imaging

As you can see on the imaging, he has a large lesion involving the anterior skull base with both intracranial and extracranial nasal components. It’s also invading into the left orbit. Given that the tumor was both intracranial and extracranial and involved the nasal sinuses, and the dura was enhancing on top of the optic nerve, we chose a combined approach—both transcranial and endoscopic transnasal.

### 1:08 Skin incision

We’ll plan a left frontal approach. And place a burr hole just below the left superior temporal line and lift off a left frontal flap to enable anterior skull base access.

### 1:28 Microsurgical resection

The dura is opened, and the tumor is identified in the anterior skull base. We cauterize the attachment and then dissect around the lateral side of the tumor and expose the carotid artery and the optic nerve. The tumor is then devascularized and debulked. We’re using an ultrasonic aspirator. We then dissect the tumor from the surrounding left frontal lobe and isolate the vascular supply and divide this after cauterization. The tumor was parasitizing the arachnoid vessels to the left frontal lobe. We then continue our dissection and cut the perforators coming from the anterior cerebral artery supply. We continue the removal of the tumor and drill out the involved skull base.

### 2:45 Left optic nerve decompression

We then cauterize the dura over the left optic nerve in anticipation of decompressing the left optic nerve and removing all the bone around the optic nerve and the involved dura. The tumor is disconnected from the left optic nerve, and we continue debulking and remove the tumor both intracranial and in the nasal cavity. Sinus mucosa is identified. The margins of the tumor are identified, and we remove normal bone to ensure complete removal.

### 3:42 Endoscopic resection

We then start our endoscopic approach. We cauterize and develop our nasoseptal flap on the right side, which is isolated from the right side of the septum. The nasal muscosa of the septum is involved with tumor.

### 4:23 Cranioendoscopic resection

And we’ll remove this in a combined fashion both endoscopically and from above. The complete margins of the tumor are then identified, and the tumor attached to the nasal septum is removed. We ensure that we come around with normal margins. The endoscopic view enables visualization of the margins of the tumor.

### 5:15 Cranioendoscopic decompression of the left orbit

We’ll then remove the medial orbital wall of the lamina papyracea and the involved periorbita. This is done in a cooperative fashion using the microscopic and the endoscopic approach. Now we’ll remove all the tumor around the left optic nerve, drill out the clinoid, and remove the superior part of the orbit just above the annulus of Zinn. Mucosa and tumor is identified in the region of the optic strut. We’ll then open up the falciform ligament and remove all the dura. The mucosa of the sphenoid is then removed to enable a landing for our nasoseptal flap. The remaining part of the involved mucosa is removed with visualization endoscopically. We drill out all the regions of involved bone.

### 6:39 Closure

Hemostasis is obtained, and we’ll now proceed with closure. We’ll place a fat graft in the region of the optic strut, and the anterior skull base dura is closed with a fascia lata graft. A dural substitute is placed over the frontal dura, and hemostasis is obtained inferiorly, and then the nasoseptal flap is placed over the fascia lata graft. After placement of the nasoseptal flap, Gelfoam pledgets are used to hold the flap in place, and a nasal splint is placed. The craniotomy is closed as is the temporalis muscle and the scalp.

### 8:00 Postoperative imaging

His postoperative MRI demonstrates complete removal of tumor.

## Acknowledgments

We thank Vance Mortimer, our video editor, for his contribution to editing the operative videos.
